# Collective Motivational Interviewing for Individuals with Drug Use Problems: A Pre-Post–Follow-Up, Uncontrolled Pilot Study

**DOI:** 10.3390/ijerph192316344

**Published:** 2022-12-06

**Authors:** Nick Tse, Samson Tse, Paul W.C. Wong

**Affiliations:** 1 Department of Social Work and Social Administration, The University of Hong Kong, Hong Kong Special Administrative Region, Hong Kong, China; 2 Department of Applied Social Sciences, HKCT Institute of Higher Education, Hong Kong Special Administrative Region, Hong Kong, China

**Keywords:** brief intervention, family therapy, concerned significant others, substance use disorders, addictive behaviours, effective intervention, counselling approach, change talk, social network support

## Abstract

Collective motivational interviewing (CMI) is a novelty motivational approach which optimises the motivational interviewing (MI) for individuals from collectivistic cultures. While MI has been empirically tested as an effective intervention for addictive disorders and has had a positive effect on facilitating lifestyle changes, CMI has retained the potency of MI as an individualistic intervention, and it further invites the social network resources to strengthen the level of motivation and cultivate a joint change partnership. This pilot study was the first clinical study of CMI to work with individuals with drug use problems (IDUPs) by involving concerned significant others (CSOs) in the three-session intervention, and the fidelity control was assessed by the Collective Motivational Interviewing Treatment Integrity (CMITI) scale. This pre-post–follow-up and uncontrolled feasibility study was conducted between 2017 and 2019, with dyads of 20 IDUPs and their CSOs. The potential impacts of CMI were examined by measures at baseline, post-intervention, and 1-month and 3-month post-intervention. All clinical sessions were audio-recorded, and four cases were randomly selected for fidelity review by two trained coders. The normality of data at the baseline was checked by a Shapiro–Wilk test. Non-parametric Wilcoxon-signed-rank test and repeated-measures ANOVA were employed for quantitative analysis. The results showed that six IDUPs had reduced drug use, and ten maintained drug abstinence with the support of CSOs, whereas four IDUPs remained unchanged or increased drug use. Overall, at the 3-month follow-up, drug use was reduced (*p* > 0.05), social support was strengthened (*p* < 0.05), and the IDUPs’ motivation for change was enhanced (*p* < 0.05). However, the small sample sizes, non-random sampling, and lack of control group may limit the generalizability and confirmation of the outcomes and of the “real effects”. This finding of the study suggests that the CMI is a feasible and acceptable therapeutic tool to motivate IDUPs with the support of CSOs to achieve mutually agreed-upon goals. Further development and evaluation with robust methodology are warranted.

## 1. Introduction

Since its inception in 1983, motivational interviewing (MI) has become a commonly adopted conversation style to elicit and strengthen motivation for change in behavioural problems within an atmosphere of acceptance and compassion [1,2]. The key feature of the MI approach is a goal-oriented and client-centred conversation style, distinct from non-directive Rogerian humanistic psychotherapy [1]. A systematic review analysed 25 peer-reviewed MI studies and showed that those cultural adaptations of MI propounded significant improvement in treatment outcomes compared to the control condition [3]. However, the efficacy was demonstrated in varying populations and diverse settings, with over 3000 randomised controlled trials [4], for example, cannabis use [5], cancer screening [6], probation [7], attention-deficit/hyperactivity disorder [8], school prevention [9] and COVID-19 vaccination hesitancy [10]. MI is predominantly delivered via a one-on-one intervention approach, then expanded as a group approach [11,12]. Adopting MI as a family intervention is still in the conceptual formulation phase, and several studies show that the involvement of concerned significant others (CSOs) would contribute to more desirable addiction treatment outcomes [13,14,15].

Collective motivational interviewing (CMI) is a recently developed form of MI by the present authors. CMI rides on a client-centric orientation, an ephemeral nature, and a change-focused counselling style, and specialised techniques of MI by expanding the MI-style practice into a social nexus paradigm [16]. The involvement of CSOs in the motivational process has lent this new realm of application. CMI focuses on the interaction process of involving multiple parties in creating joint change partnerships [16]. The six-process model (now refers to six tasks) includes engagement, focus, evocation, and plan to cultivate clients’ and CSOs’ changing momentum for the individual sessions. Two additional tasks, “join” and “collection”, were developed to foster collective motivation in the conjoint session. For details regarding CMI, see the earlier publication [16]. The current article reports the preliminary results of a feasibility examination of CMI to address individuals with drug use problems (IDUPs) within the social nexus paradigm.

MI incorporated with CSOs traces back to three large MI-based research projects: Project MATCH [17], COMBINE [18], and UKATT [19]. These studies all advocated the involvement of CSOs and recognised that their participation could help clients make decisions about drinking habits. Five major critiques were raised in these projects: (a) Position hierarchy: Rather than clients and CSOs working as a cohesive partnership, CSOs played an ancillary role, giving information and acting as a monitor for the client’s mediation compliance and treatment attendance; however, this may trigger defensiveness in the clients and may undermine their intrinsic motivation. (b) Presumption of the readiness and supportive attitudes of CSOs: It remains unclear how these projects prepared CSOs to be involved in the conjoint session(s). The counsellor–CSO therapeutic interactions and intervention strategies were not clearly documented. The absence of the prior preparation of clients and CSOs may run the risk of their prematurely entering treatment. The proper preparation of clients and CSOs for the conjoint sessions could serve to assess and detect any sensitive issues and attitudes of a CSO regarding the client’s motivation to change. (c) Overlooking the synergic potency of the conjoint session: The preparation of family members or CSOs before the session and the adoption of clear meeting rules and guiding principles during the session are of paramount importance to ensure meaningful and constructive dialogue and a safe process for all individuals concerned. (d) Selection criteria of CSOs were absent. (e) Involvement of CSOs was optional.

Several scholarly works subsequently attempted to develop and apply MI as a family intervention but stayed at the conceptual level of evolution. Steinglass [20] combined family systems with motivational enhancement therapy, which describes the conceptual framework and the potential application of MI from the systemic family perspective, but no relevant clinical trial or case study has been reported so far. Lloyd-Hazlett, Honderich and Heyward [21] integrated MI and family counselling, discussed the potential application at the system level, and highlighted the importance of a therapeutic alliance in the family system and in goal-setting. Huang, et al. [22] suggested that family-integrated MI interventions were more effective than health education in changing smoking habits, by encouraging communication and fostering positive dialogue between smokers and their families; as a result, the lower-motivated smokers were more likely to change their smoking behaviours. Douaihy, Kelly and Gold [23] advocated for MI to be incorporated as extra social support from family, aiming at evoking clients’ motivation for change. However, it is ambiguous in that applying only the conceptual framework of MI in the family is insufficient to support the feasibility and efficacy of MI with respect to the involvement of CSOs. The question of “not knowing” how to prepare and mobilise the social support to facilitate change is still unanswered in motivational counselling. To answer this question, consolidating theoretical efforts to tailor a precision therapeutic for motivating the client by preparing the readiness of the CSOs joining a synergistic partnership is a more promising approach to fostering changing momentum for the clients by mobilising the support of social network resources. The aim of the present study is to use a pre-post–follow-up within a group pilot study to investigate, by involving CSOs, the feasibility and potential outcomes of CMI as an intervention for IDUPs.

## 2. Materials and Methods

### 2.1. Participants

Research participants were individuals affected by drug use problems and referred by the community-based drug counselling facilities in Hong Kong. Inclusion criteria for the IDUPs included (a) the severity of drug use at a low level or above as screened by the Drug Abuse Screening Test (DAST) [24], (b) low motivation to seek rehabilitation, not having received regular and structured drug counselling treatment in the past 3 months; and (c) age above 18 years. The inclusion criteria of CSOs included (a) a close relationship with the IDUPs (not limited to family members), (b) nomination by the IDUPs, (c) self-reported no drug use in the last 12 months, and (d) age above 18 years. Exclusion criteria for both IDUPs and CSOs were (a) aggressive or suicidal behaviour in an acute phase, (b) life-threatening medical conditions, (c) non-provision or unwillingness to provide written informed consent to participate in the study, and (d) facing impending incarceration. 

### 2.2. Sample Size

The present study examined the feasibility of the newly developed CMI and was not a full-scale effectiveness study using an RCT. This study evaluated the feasibility of recruitment, retention, assessment instruments and procedures, the implementation of a new intervention approach [25], and preliminary outcomes of CMI. Estimating the sample size using the power is usually not pragmatic or necessary in a pilot trial [26]. In the current pilot study, a single group design examines the efficacy of intervention; as long as population effect sizes are moderate to larger, the sample size between 20 and 25 will probably be sufficient [27]. Thus, 20 pairs of IDUPs and CSOs were recruited to participate in this novelty intervention pilot study [28]. 

### 2.3. Intervention

The consenting participants were invited to participate in the baseline assessment (T0) in their counselling room with an independent fieldworker who was responsible for data collection and logistics with the referral’s agency. After completing the brief assessment, the IDUPs and CSOs were invited to attend the first two sessions separately (each lasting for 40–60 min) before participating together in the third conjoint session (lasting approximately 60–75 min). The individual-MI sessions for the IDUP and CSO aim to enhance their motivation, instilling hope and shaping positive attitudes and language use in the subsequent conjoint session (manual available upon request) [29]. The implementation of CMI intervention was conducted by the founding researcher (N.T.) of CMI, who is a registered social worker by training, with a doctoral degree, and is a certified MI trainer accredited by the MI network of trainers (MINT). In addition, the independent fieldworker conducted an in-person follow-up interview with participants (both the IDUPs and CSOs) at the time points of immediate after-intervention (T1), 1-month follow-up (T2) and 3-month follow-up (T3). A travelling allowance was given to the IDUPs and their CSOs in the form of supermarket coupons worth HKD$100 (≈USD$12.8) for the completion of baseline and post-intervention; for participation in each interview, coupons worth HKD$50 (≈USD$6.4) were given. The present study was approved by the Human Research Ethics Committee for Non-Clinical Faculties at the University of Hong Kong (HRECNCF Ref. no. EA1606045).

### 2.4. Measures

#### 2.4.1. Drug Abuse Screening Test

The Drug Abuse Screening Test (DAST) is a unidimensional screening tool consisting of 20 items designed to detect the severity of the drug addiction problem and to evaluate treatments [24]. The test is a self-administrated questionnaire with good validity and reliability with a Cronbach’s α of 0.95 [24]. This instrument is one of a few instruments for clinical and non-clinical assessment of the severity of drug use and associated problems, with reported results close to those for the diagnosis of drug dependence in DSM-III. The summarised scores were categorised into five levels of drug use severity and provide a corresponding action/treatment according to the American Society of Addiction Medicine Placement Criteria: 0 (no drug abuse problem; no action or treatment is needed), 1 to 5 (low level; brief counselling is recommended), 6 to 10 (intermediate level [similar to DSM-III criteria]; intensive-outpatient service is suggested), 11 to 15 (substantial level; intensive follow-up is required), and 16 to 20 (severe level; intensive follow-up is proposed).

#### 2.4.2. Timeline Follow-Back Interview

Timeline Follow-back (TLFB) interview is a calendar-based measure of self-reported drug use, including the amount and frequency of drug use. The TLFB method measures the self-reported change in the frequency of drug use from baseline and post-intervention to post-intervention follow-up (i.e., 1-month follow-up interview and 3-month follow-up interview) [30]. This instrument has been reported to have good reliability in measuring the frequency of drug use. The psychometric property of this instrument was reported to have high reliability and reached interclass correlation coefficient values of between 0.70 and 0.94 (all *p* < 0.001) [31]. The use of TLFB makes gathering information on drug use easier because it limits the need for biological testing [32]. 

#### 2.4.3. Contemplation Ladder

A “contemplation ladder” can be employed to measure the motivation to abstain from drug use based on a single brief option of 11 rungs and five types of statements [33,34]. The ladder was derived from a transtheoretical model of change, which asserts a five-stage linear model of change: pre-contemplation, contemplation, preparation, action, and maintenance [35]. The instrument is rated on a scale from 0 to 10, with each point representing a specific statement showing a corresponding stage of change (i.e., level of motivation to change). The options are 0 to 3 (representing the pre-contemplation stage), 4 to 6 (matching the contemplation stage), 7 and 8 (corresponding to the preparation stage), and 9 and 10 (equal to the action and maintenance stage). The ladder has been used in smoking cessation studies, which exhibited strong reliability and validity with strong intercorrelations (Pearson’s r = 0.82–0.98) [36]. The contemplation ladder has also been shown to have good discriminant, convergent, and predictive validity in adults with substance use disorder [37]. 

#### 2.4.4. Multidimensional Scales of Perceived Social Support

The multidimensional scale of perceived social support (MSPSS) is a self-administered instrument designed to measure the level of social support perceived by the client. It comprises 12 items scored on a 7-point Likert scale ranging from 1 (very strongly disagree) to 7 (very strongly agree). The three sub-categories of support were identified as family, friends, and significant other with strong factorial validity. This instrument demonstrated good internal and test-retest reliability as well as moderate construct validity [38]. The Cantonese version (the MSPSS-C scale) has been reported to have an internal consistency coefficient reaching a Cronbach’s α of 0.89 [39]. The mean scores ranged from below 3 (low social support), between 3 and 5 (moderate social support), and between 5.1 and 7 (high social support). 

#### 2.4.5. Treatment Integrity of Collective Motivational Interviewing

The treatment integrity of CMI was monitored by a modified coding system, which was reviewed by two experienced MI trainers and advised by one expert coder of MI. The Collective Motivational Interviewing Treatment Integrity (CMITI) coding manual [40] incorporated the original MI Treatment Integrity (MITI 4.2.1) [41,42], which performed to the individual MI session for the client and CSO and the collective tenets were monitored by the additional codes, including global rating: *Technical components* added “cultivating CSO support language” (CCSL) and “softening CSO indifferent language” (SCIL); *Relational components* added “neutrality” (N), “mutual empathy” (ME) and “consensus” (C); behaviour counts added “affirm to client” (AC) and “affirm to CSO” (ACSO); and interaction scores included “support” (CSO-S), “contemptuousness” (CSO-C) and “partnership” (between client and CSO). The CMITI manual underwent the processes of peer review and MI-expert consultation to ensure that the coding items were relevant and reflected the essence of CMI to ensure the face-validity of the coding instrument. Two experienced MI coders were trained by N.T. to use the coding system, and they were required to pass the coding competency assessment in order to be involved in the integrity examination process. All 60 interview sessions were audio-recorded. The quality of intervention was ascertained by selecting 20% of sessions for coding with the CMITI coding manual.

### 2.5. Data Analysis

Data were collected at baseline (T0), post-intervention (T1), 1-month follow-up (T2), and 3-month follow-up (T3). For the quantitative analysis, the IBM SPSS Statistics for Winders, Version 24.0 [43] was employed. Participants’ demographic characteristics were analysed by descriptive analyses. We used the linear regression type of multiple imputation method to impute the missing values for the outcome variables at T1 (10%, *n* = 2 of missing data) and 3-month follow-up (20%, *n* = 4 of missing data) on the outcome measures of time-line follow-back, contemplation ladder and multidimensional scale of perceived social support [44]. All outcome measures underwent normality testing (i.e., *p*-value of Shapiro-Wilk < 0.05, then the null hypothesis was rejected, implying that the variable was not normally distributed). If a significant *p*-value was obtained, parametric ANOVA was used to test the mean of the measures. Otherwise, non-parametric tests were used to track the changes across the four time points. Quantitative data were analysed by the non-parametric Fisher’s exact test for categorical variables and the Friedman test for continuous variables. For the determination of effect size, the Wilcoxon signed-rank test, including Bonferroni–Holm correlations, was used. The thresholds for the effect size interpretation were *r* ≤ 0.3 (small effect size), ≤0.5 (medium effect size), and >0.5 (large effect size) [45]. Significance was determined at a two-tailed *p* < 0.05. 

## 3. Results

### 3.1. Participant Profiles

In this trial, seven drug counselling centres referred 20 pairs of IDUPs (*n* = 20) and their CSOs (*n* = 20). Participant demographics are found in Table 1. The gender ratio of the IDUPs was 3:1 (75% males), similar to the ratio of reported drug users (80% males and 20% females) in Hong Kong in 2018 [46]. Participant CSOs were 80% female versus 20% male. The majority of the IDUPs were aged between 18 and 40 years old (80%), and half of them were never married; 90% of the IDUPs were living with family members. Educational attainment was comparatively low; 90% did not have tertiary education, with half of the IDUPs being unemployed in the past month. Median income was low (85%) compared to the median household monthly income of HKD$28,700 (≈USD$3680) in Hong Kong in 2019 [47], and 35% received Comprehensive Social Security Assistance (CSSA), a government allowance that serves as a safety net to support the basic living needs of those who are in financial difficulty [48].

The severity of drug use and the psychiatric condition of clients is also reported in Table 1, showing that 85% of the IDUPs reached an intermediate (15%) and substantial (70%) level of drug abuse (likely meeting DSM criteria) measured by the DAST. Average years of drug use was 15.2 (*SD* = 10.5). The main types of drug use ranged from amphetamine (40%), cough medicine (25%), heroin (15%), cocaine (10%), and midazolam (10%). Self-reporting on receiving psychiatric treatment was 70% (lifetime) and 45% (currently), respectively. Of the clients, 40% self-reported that they had been diagnosed with psychiatric disorders by psychiatrists, including depression (30%), bipolar disorder (5%), and drug-induced psychosis (5%). 

### 3.2. Baseline Measures

Of the clients, 85% fit the DSM criteria or reached a substantial level of drug addiction as measured by DAST (see Table 1). According to the American Society of Addiction Medicine guidelines, these clients should be matched with intensive-care drug treatments, such as intensive out-patient or in-patient services. Such clients participating in the current project were still in the community and using drugs. In line with the TLFB, the mean of percentage days of using drugs (3 months before participating in CMI) was *M* = 44.32%, *SD* = 36.20. The contemplation ladder mean score was *M* = 6.55 out of 10, *SD* = 2.04, indicating clients at baseline were at the contemplation stage of change and were considering/struggling at the ambivalent state of continuous drug use without taking any action. In addition, the mean score of MSPSS *M* = 4.58 out of 7 (*SD* = 1.59) reflected a low level of social support. At the baseline, characteristics of clients fitted the inclusion criteria of the present study including severe drug use problems, regular drug use in the prior 3 months, level of motivation to change, and level of social support. 

### 3.3. Outcome Measures

**Figure 1:**
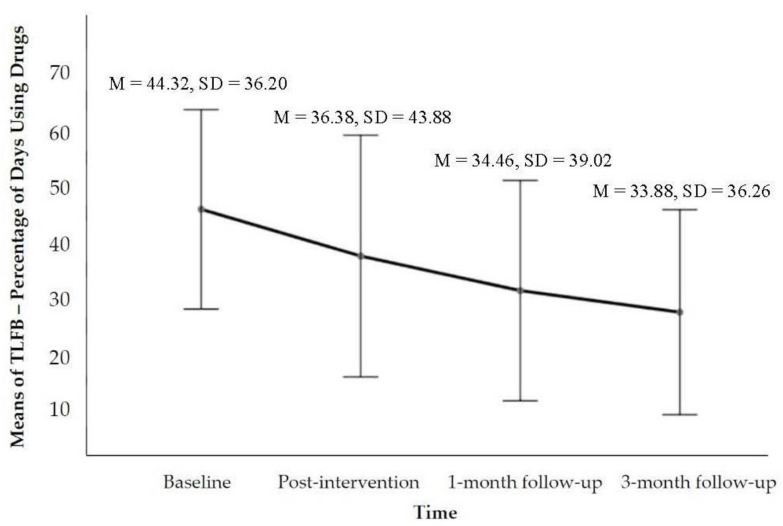
Estimated marginal means plot showing the TLFB changes as percentages of days across the four time points.

**Figure 2:**
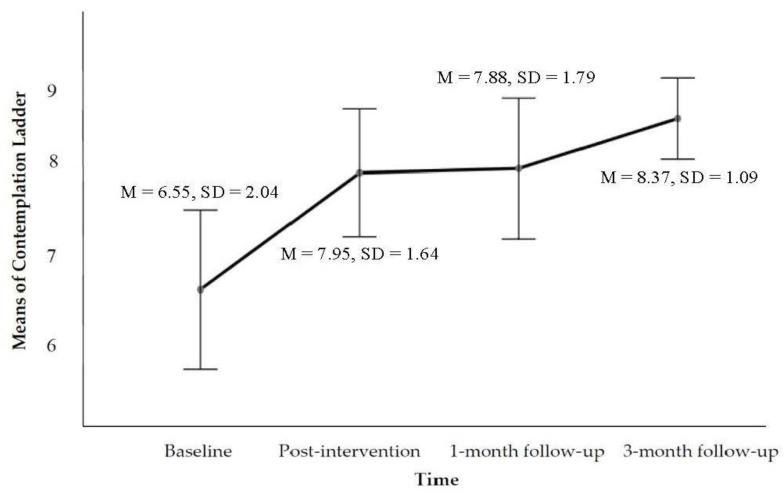
Estimated marginal means plot showing the change of contemplation ladder scores across the four time points.

**Figure 3:**
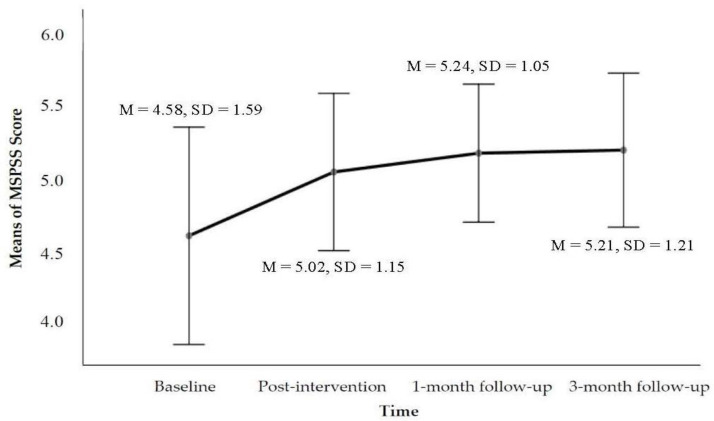
Estimated marginal means plot showing the changes of MSPSS scores across the four time points.

The CMI was designed primarily to achieve three outcomes in the present study: reducing drug use (see Figure 1), enhancing motivation to change (see Figure 2), and strengthening social support (see Figure 3). Compared to baseline at the 3-month follow-up, the mean score of TLFB was *M* = 33.88%, *SD* = 36.26, and *p* = 0.391. The Wilcoxon signed-rank test revealed a statistically significant reduction in the percentage of days using drugs at the 3-month follow-up following participation in the CMI intervention, namely *z* = −0.86, *p* > 0.05, and *r* = −0.14. The median score of TLFB as a percentage of days using drugs decreased from the baseline (*Mdn* = 42.2) to the 3-month follow-up (*Mdn* = 28.54) post CMI intervention. The abstinence outcome at the 3-month follow-up reached 50% (including 10% of the IDUPs who took action to enter voluntary residential drug-rehabilitation programmes, which were planned in the counselling process). Apart from total abstinence, 30% of the IDUPs reported reduction, and 20% reported an increase in the frequency of drug use (see Table 2). Regarding the tracking of the changes of the contemplation ladder, the result of the general linear model (repeated measures ANOVA) indicated a statistically significant time effect on the level of motivation across the four-time points, Wilks’ lambda = 0.450, *F*(3, 17) = 6.92, *p* = 0.003, and η^2^ = 0.550. Using the same test for the MSPSS, a statistically significant time effect on the level of social support across the four time points was found: Greenhouse–Geisser *F*(2.528, 32.776) = 3.810, *p* = 0.021, and η^2^ = 0.167 (see Figure 3). The thresholds of the effect size followed the guidelines proposed by Cohen [45], which were 0.01 = small, 0.06 = moderate, and 0.14 = large effects. The obtained partial eta squared (η^2^) suggested a very large effect size.

### 3.4. Fidelity Control

A coding process using the CMITI manual was applied to the present study to check the adherence to CMI practice and to ensure that the outcomes truly reflected the impact of the intervention [49]. This should improve transparency and determine the quality of the intervention [50]. A total of 60 CMI sessions, completed in 20 cases, were audiotaped for coding. Two coders signed a confidentiality agreement before the coding process. Four cases (*n* = 4, 20%) were randomly selected using random.org [51], giving a total of 12 sessions. The randomly selected segments from the interview sessions were coded following the randomisation guidelines suggested by an experienced MI coder. The disagreement on the coding results between the two coders was discussed and resolved within the fidelity control panel (consisting of two coders and the CMITI developer) to come up with a consensus. The coding result was shown below (see Table 3).

## 4. Discussion

The results of this feasibility study demonstrate that the CMI has the potential to be applied to IDUPs and their CSOs. Preliminary results show positive effects on reducing the frequency of drug use, strengthening the motivation for change and enhancing social support. The study fills one of the most overlooked gaps in the field of counselling and psychotherapy, namely that of preparing the readiness of both the CSOs and the clients participating in the therapeutic process and in turn effecting joint changes. However, the present feasibility study cannot verify the causal inferences between CMI and the drug treatment outcomes and a randomised controlled trial is required to confirm these results.

It is encouraging to observe a 100% treatment completion rate in the study, which seems to show that CMI was well accepted by all pairs of clients and CSOs. We found that the involvement of CSOs can potentially contribute to the positive trends in the outcome indicators, even if research participants have a long drug use history and co-morbid conditions. At the 3-month follow-up, 80% of clients showed some reduction in drug use, including abstinence in 50% (10 out of 20 cases). Of the abstinence cases, 70% were in the community and 30% in residential settings (see Table 2). The preliminary results show that the research participants not only strengthened their intention to change but also initiated action-taking behaviours; motivation has long been a crucial component of drug treatments [52,53,54]. The clients’ motivation to change is closely associated with the retention rate and with drug treatment outcomes [55,56,57]. The major change in outcome was observed in motivation level, which obtained a large effect size. This effect is in conjunction with the goal of CMI, namely, to enhance client’s motivation for change with the support of CSOs.

Recovery from drug addiction can be a very lonely process, and for many recovering individuals [58], relapse is common when the social support network is weak [59,60,61]. CMI is a psycholinguistic motivational tool designed to go beyond so as to shape the individual dialogue, basing it on the original MI, but including both of the multiple-involved parties’ language and expression in the counselling process [16]. The original design of CMI is prone to evoke the client’s change talk and the CSO’s support language, while at the same time softening client’s sustain talk and the CSO’s indifferent language during the motivational process. Future studies that adopt a psycholinguistic analysis of the narratives of the recovering individuals and their CSOs during the intervention will further enhance our understanding of the language being used during the process. Moreover, process evaluation is also encouraged so as to unfold the active ingredients and the linguistic interaction during the CMI. On the other hand, disruptive social relationships can also trigger a relapse of drug use [62,63,64]. Therefore, precautions should be taken when initiating social support, for example, by helping the client and CSOs develop healthy activities and mutual interests for a positive and healthy social network [65,66,67].

### 4.1. Limitations

The small sample sizes may limit the statistical power to detect statistically significant results in some variables, limiting the generalizability of the results. The absence of sampling randomisation might lead to sampling bias. With no control group, it is uncertain whether the positive findings were attributed to the intervention or to other confounders, for example, time effects, practice effects with repeated assessments, or placebo effects. The preliminary evidence lays the groundwork for a large-scale trial with more sophisticated and robust research methodologies to further examine the effectiveness of CMI. Although self-reporting among the drug use population has long been known to be sufficiently reliable, valid, and useful [68,69], some challenge the trustworthiness and reliability of self-reported data in drug treatment research [70].

### 4.2. Implications

Having acknowledged these limitations, the findings of this feasibility study shed light on several insights that support both scaling up the clinical trial with robust methodology and the dissemination of CMI for further development. First, this study displays that CMI is feasible and acceptable, and it could project a clinically effective intervention in the real-life clinical settings for IDUPs. Notably, a trend of improvement in the outcome indicators was observed with research participants experiencing chronic drug use and complex comorbidity. Furthermore, a recent systematic review shows that there is currently no evidence-based treatment for internet gaming disorder (IGD), despite well-designed clinical trials [71]. It is important to involve parents in the treatment of adolescents with IGD [72]. As the development of addictive disorders takes time, early identification and treatment would be beneficial to ease addictive disorders or delay their development. The development of CMI is still in its infancy. Time is still needed to both consolidate the intervention and to fine-tune the intervention protocol to mature this approach (approach level). Further, investigations using robust scientific research methods are imperative to examine the efficacy and effectiveness of CMI on other addictive behaviours such as IGD, and life-style problems, such as weight control and unhealthy sleep habits.

## 5. Conclusions

The encouraging preliminary results generated from the 20 IDUPs and 20 CSOs demonstrated the applicability of CMI as an intervention for those with drug use problems. The brief three-session CMI intervention with CSO involvement was probably able to both reduce the clients’ frequency of drug use and enhance their motivation to change with the support of CSOs. In addition, because behavioural addictions are causing much concern across the globe, impacting individuals, families, and communities, the addiction field urgently needs to develop an evidence-based, low-threshold, low-intensity counselling approach for behavioural addictions. CMI shows promise as a useful tool for drug addiction and could potentially be extended to other behavioural addictions. 

## Figures and Tables

**Table 1:** Sociodemographic characteristics and drug use background of participants at baseline.

Baseline Characteristics		
	*n*	%
Clients (IDUPs)—Individuals with Drug Use Problems		
Gender		
Male	15	75
Female	5	25
Age (groups in years)		
18–25	3	15
26–30	6	30
31–35	3	15
36–40	4	20
41–45	1	5
46–50	1	5
51–55	2	10
Education level		
Primary education (P.1 to P.6)	1	5
Junior secondary education (S. 1 to 3)	8	40
Senior secondary education (S. 4 to 7)	9	45
Tertiary education (associate degree or above)	2	10
Employment/Study status		
Full-time (at least 44 h per week)	6	30
Part-time (less than 44 h per week)	2	10
Unemployed	10	50
Full-time study	2	10
Marital status (% of total sample)		
Single, never married	10	50
Married/cohabiting	7	35
Divorced	1	5
Widowed	2	10
Living with family members		
Yes	18	90
No	2	10
Personal income (monthly, HKD)		
No income	7	35
$4999 or below	5	25
$5000–$9999	3	15
$10,000–$19,999	2	10
$20,000–$29,999	2	10
$30,000–$39,999	1	5
Comprehensive Social Security Assistance (CSSA)		
Yes	7	35
No	13	65
Had received psychiatric treatment		
Yes	14	70
No	6	30
Currently receiving psychiatric treatment		
Yes	9	45
No	11	55
Drug Abuse Screening Test (DAST)		
Low	3	15
Intermediate (likely meeting DSM criteria)	3	15
Substantial	14	70
History of drug use (years)		
Below 5	3	15
5 to 10	6	30
11 to 20	3	15
21 to 30	4	20
31 or above	4	20
Main types of drug use		
Heroin	3	15
Cocaine	2	10
Amphetamine	8	40
Cough medicine	5	25
Midazolam	2	10
Self-reported that they had been diagnosed with psychiatric disorders by a psychiatrist		
Depression	6	30
Bipolar	1	5
Drug-induced psychosis	1	5
Unsure (forgotten by respondents)	6	30
No	6	30
Concerned Significant Others (CSOs) of IDUPs		
Gender		
Male	3	15
Female	17	85
Relationship with client		
Mother	6	30
Father	1	5
Older sister	4	20
Wife	4	20
Husband	1	5
Daughter	2	10
Close friend (Male)	2	10

**Table 2:** Frequency table changes in drug use and abstinence at 3-month follow-up (*N* = 20).

Drug Use Status at T3	*N*	*%*
Stayed in community after CMI		
Abstinence-C (A)	8	40
Reduced in drug use frequency	6	30
Increased in drug use frequency	4	20
Entered voluntary residential drug rehabilitation programmes Abstinence-R (B)	2	10
Total abstinence in the present study (A+B)	10	50

Note. Abstinence-C = stayed in the community; Abstinence-R = entered voluntary residential drug rehabilitation programmes.

**Table 3:** Descriptive results of CMI coding (four cases randomly selected from the total sample).

	Quality of CMI in the Present Study			The Thresholds of CMITI	
	Session 1	Session 2	Session 3	Fair	Good
Relational	4.8	4.3	4.5	3.5	4.0
Technical	4.5	4.3	4.5	3.0	4.0
%CR	61.3%	57.1%	64.3%	40%	50%
R: Q	0.9:1	1.9:1	1.4:1	1:1	2:1

Note: %CR represents the number of complex reflections divided by the total number of complex and simple reflections. The ratio of R:Q represents the number of simple plus complex reflections divided by the number of questions.

## Data Availability

Not applicable.
